# Subcutaneous hemangioma on nasal dorsum: a case report

**DOI:** 10.1186/s13256-020-02443-4

**Published:** 2020-08-13

**Authors:** Hamsu Kadriyan, Muhammad Alfian Sulaksana, Didit Yudhanto, I. Gusti Ayu Trisna Aryani, Eka Arie Yuliani, Nurul Endah Ardianti, Moh. Suprayogi, Fathul Djannah

**Affiliations:** 1 Department of ENT-HNS, Faculty of Medicine, Mataram University/West Nusa Tenggara Regional Hospital, Jl. Pemuda No. 37, Mataram, West Nusa Tenggara 83125 Indonesia; 2 Department of Pathology and Anatomical Sciences, Faculty of Medicine, Mataram University/West Nusa Tenggara Regional Hospital, Jl. Pemuda No. 37, Mataram, West Nusa Tenggara 83125 Indonesia

**Keywords:** Subcutaneous hemangioma, Pediatric, Adult, Remote area, Elliptical approach

## Abstract

**Introduction:**

Hemangioma is a benign tumor made up of blood vessels and typically occurs as a slightly elevated purplish or reddish area of skin. Hemangioma is mostly found superficially; subcutaneous hemangioma in the nasal dorsum is rare.

**Case presentation:**

In West Nusa Tenggara Regional Hospital, the authors found two cases of subcutaneous hemangioma in patients of very different ages. The first patient was a 2-year-old Sasak girl, and the other was a 40-year-old Sasak man. The pediatric patient was treated with an elliptical approach, whereas the adult patient was treated with lateral rhinotomy extended by an elliptical approach to remove the hemangioma and ligate the feeding arteries. After surgery, the adult patient was followed up for 5 months, whereas the pediatric patient was followed up for 3 months. The results for both patients were good, with minimal scar formation.

**Conclusion:**

Despite the limitations of technology and human resources in a remote area of Indonesia, the surgical approach used in these cases produced good outcomes for both patients.

## Introduction

Vascular anomalies are congenital lesions of abnormal vascular development. There are two major groups of vascular abnormalities, including vascular malformation, which is a local defect in vascular morphogenesis, and vascular tumor (hemangioma), which is cellular hyperplasia [1]. Hemangioma usually occurs in pediatric patients; however, adult cases are also possible. A study in Israel identified a majority of female patients among ten patients with nasal tip pediatric hemangioma [2]. Hemangioma is common in adult males in any age group. It was found that 60% of hemangiomas in adults were located in the head and neck region and involved the skin, subcutaneous tissues, tongue, nasal mucosa, oral cavity, larynx, and salivary glands [3]. The rest of the hemangiomas occurred in the trunk (25%) and extremities (15%) [4].

Approximately 15.8% of facial hemangioma cases occur in the nose. A case of nasal dorsum hemangioma was reported by Waner *et al.* [5], who found 44 patients in their series. They treated the patients with several approaches, such as external rhinoplasty, modified unit, or elliptical and midline elliptical. They performed two surgeries with the midline elliptical and elliptical approaches; furthermore, the results were varied from unsatisfactory to good. Similar to the other technique, the results also varied between unsatisfactory, improvement, good, very good, or satisfactory [5].

Deep or subcutaneous hemangioma is considered rare, with only 15% of cases being subcutaneous hemangioma. The majority of hemangioma cases were superficial hemangioma and mixed hemangioma, with the proportions of cases being 50–60% and 25–35%, respectively [6]. Subcutaneous hemangioma appears as a warm subcutaneous mass because of the tumor proliferation localized in the deeper portion of the dermis or subcutaneously. During the proliferation phase, the tumor has high-flow blood supply by arteries; hence, physical examination may reveal the presence of bruits. In smaller subcutaneous hemangioma, the skin may appear normal with inconspicuous changes of the skin, such as telangiectasia or dilated veins [1].

Pitanguy *et al.* advocated a midline elliptical incision, which can give good functional results and preserve the nasal contours but leaves a rather obvious midline scar on the nasal dorsum [7]. An elliptical midline incision gives easy access to the nasal dorsum, but in the lateral alar extension and columella area, this approach is not comparable to other modified subunit incisions [5]. Simic *et al.* performed three different surgical techniques for nasal dorsum hemangioma: open rhinotomy, lenticular excision, and circular excision. They reported that open rhinotomy provided a better outcome than circular excision and lenticular excision [8].

## Case presentation

The authors found two cases of hemangioma on the nasal dorsum in patients of very different ages in West Nusa Tenggara Regional Hospital. The first patient was a 2-year-old Sasak girl, and the other was a 40-year-old Sasak man.

**Fig. 1:**
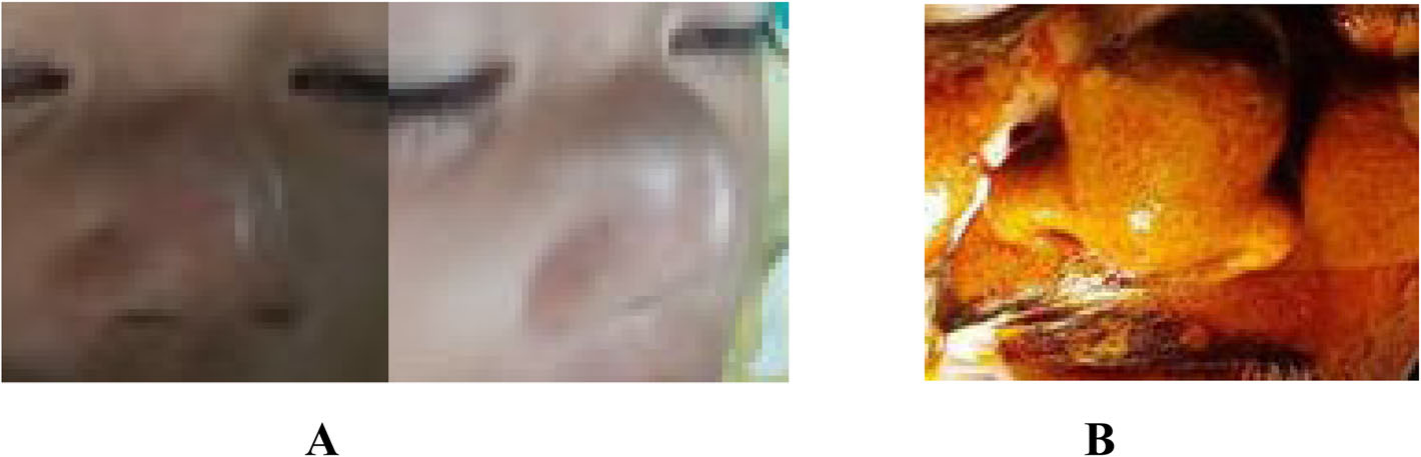
Patients before surgery. **a** Patient 1. **b** Patient 2

**Fig. 2:**
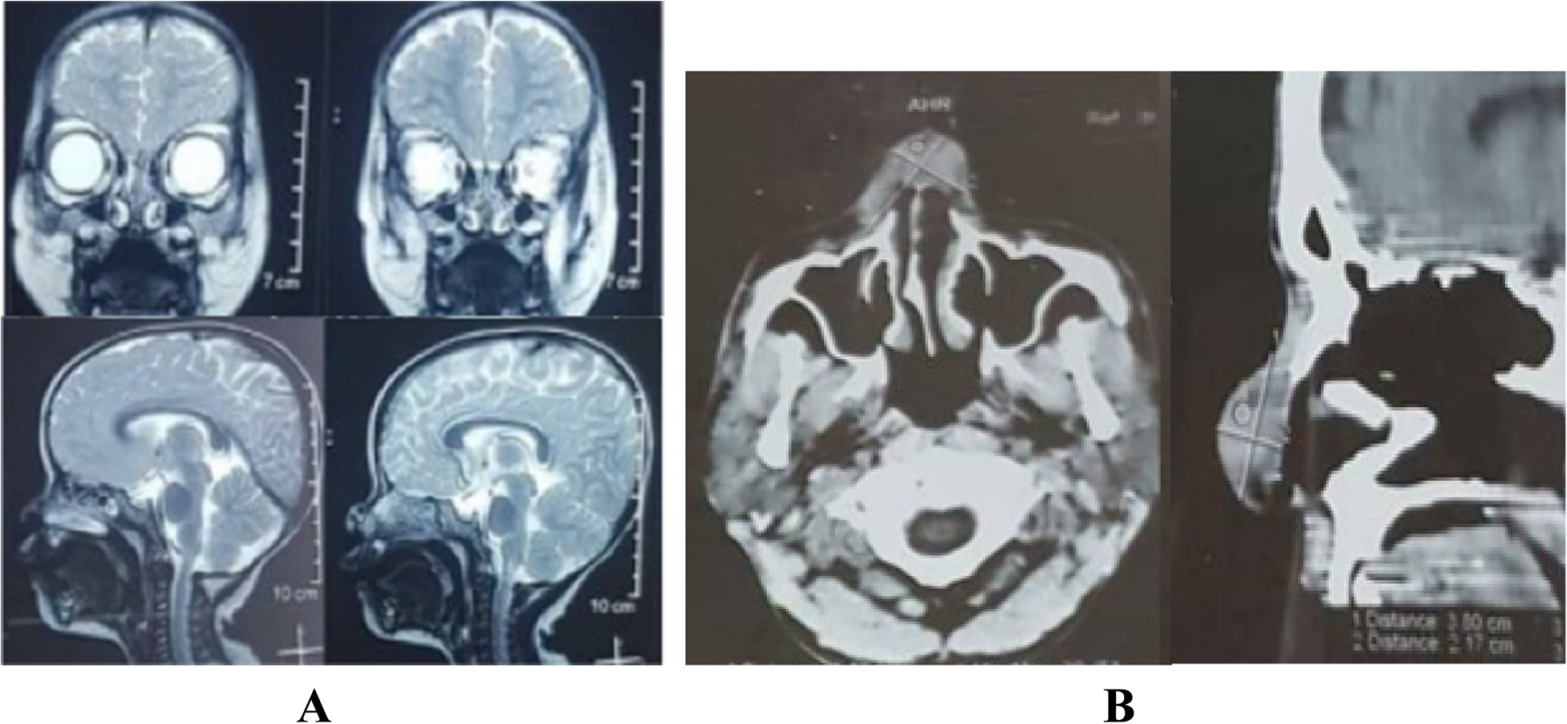
Computed tomographic scans of the patients. **a** Patient 1. **b** Patient 2

**Fig. 3:**
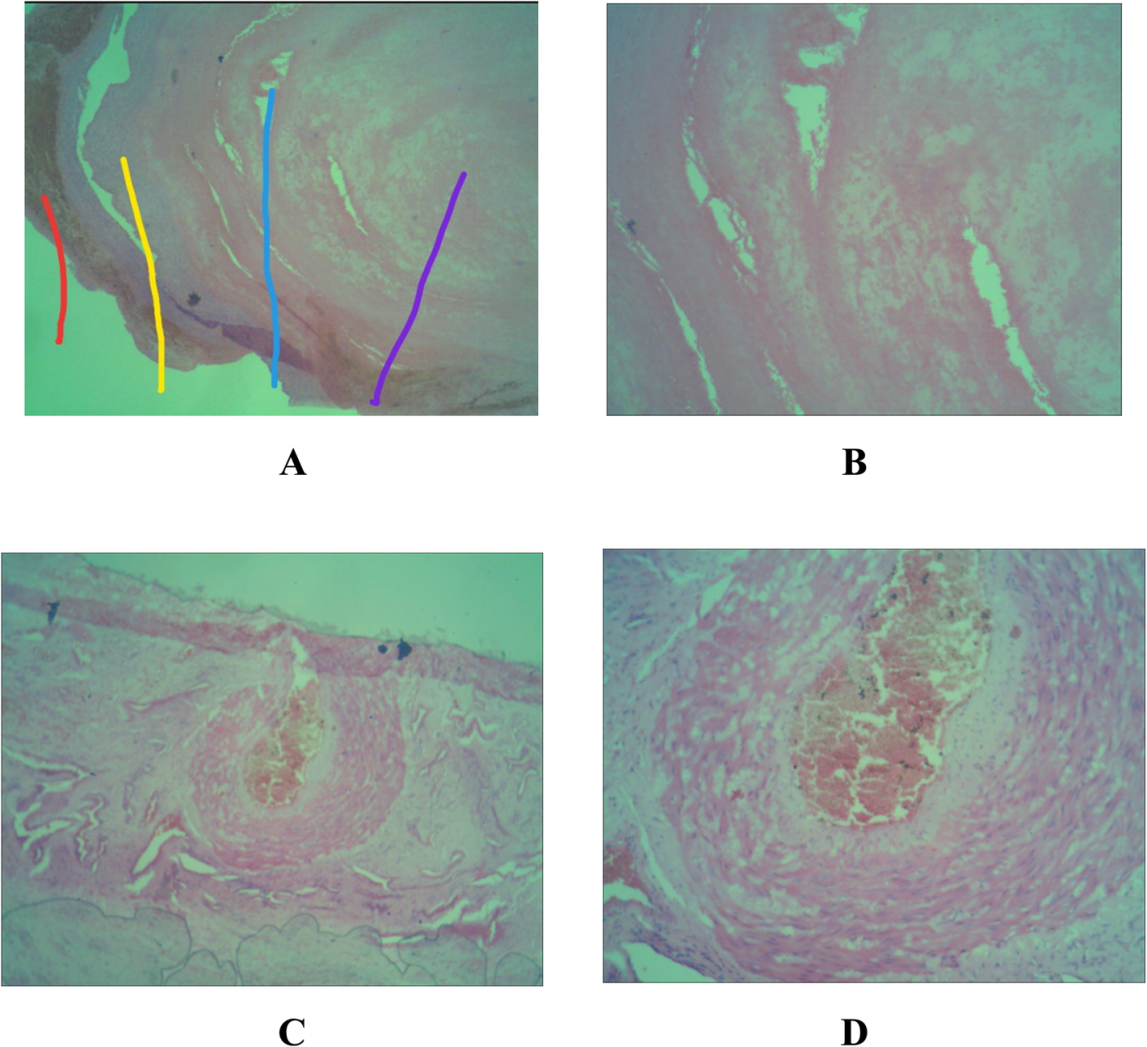
Histopathologic findings. The *red line* indicates previous bleeding; the *yellow line* indicates the tumor epithelium; the *blue line* indicates the tumor with a big vessel; and the *purple line* indicates the tumor with a small vessel. **a** and **b** Patient 1. **c** and **d** Patient 2. Original magnification 40× in **a** and **c**, 100× in **b** and **d**

**Fig. 4:**
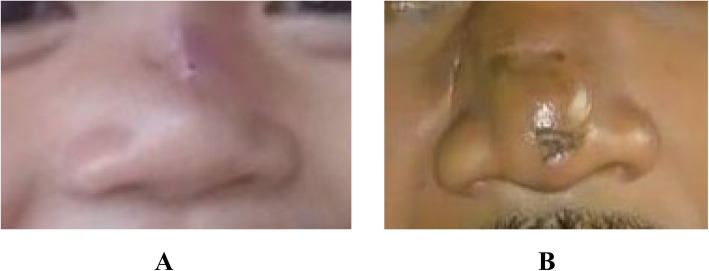
Follow-up was conducted at 3 months and 5 months after surgery, respectively, in patient 1 (**a**) and patient 2 (**b**)

Patient 1 was a 2-year-old Sasak girl who had a bulky soft tissue mass on the nasal dorsum of 3 months’ duration (Fig. 1a). The mass had grown bigger within the past month. She had no history of trauma on the face or any sign of bleeding. She had no history of treatment, either pharmacologic or surgical. Her physical examination revealed no reddish patch on the surrounding skin. During palpation, a soft mass was found with an unclear margin and arterial pulsation on the mass. A computed tomographic (CT) scan showed a soft tissue mass on the surface of the nasal dorsum without any damage in the adjacent tissue or bone (Fig. 2a). On the basis of those results, the authors concluded that this was a suspected case of subcutaneous hemangioma. In this case of suspected hemangioma, a biopsy was not recommended, owing to its risk of bleeding. This patient was treated with surgery through an elliptical approach. During the surgery, the amount of bleeding was minimal. Histopathologic findings after surgery showed a tissue covered by squamous epithelial cells and, on its inside, a proliferation of large arteries containing erythrocytes (Fig. 3a and b). This result indicated that this case was one of cavernous hemangioma. Follow-up 3 months after surgery revealed that there was a mild scar in the location of the incision (Fig. 4a). According to an interview with the patient’s parents, they were satisfied with the result of surgery and finally concluded the outcome was good on the basis of the scale of very poor, poor, fair, good, very good.

Patient 2 was a 40-year-old Sasak man known to have a mass for more than 2 years. He complained of a bulky soft mass on the nasal dorsum (Fig. 1b). The mass was growing slowly and was painless. The patient had no history of bleeding or disturbance caused by nasal blockage. He had no history of previous pharmacologic or surgical treatment. He had no reddish patch on the surrounding skin during the inspection; however, he had a mass on the nasal dorsum with arterial pulsation during palpation. Therefore, this case was concluded to be one of subcutaneous hemangioma. To support the diagnosis, imaging of the face was done with a CT scan; furthermore, a soft tissue mass on the surface of the nasal dorsum was found without any damage in the adjacent tissue or bone (Fig. 2b). A preoperative biopsy was not performed, owing to the risk of bleeding. In this case, lateral rhinotomy with extension of the elliptical approach was performed to remove the hemangioma and ligate the feeding arteries. During the surgery, the amount of bleeding was moderate. The postoperative histopathologic examination found tissue with large artery proliferation covered by endothelium and containing erythrocytes (Fig. 3c and d). This result supported the case as one of cavernous hemangioma. Five months after surgery, the wound was healed with minimal scarring (Fig. 4b). According to the patient interview with similar criteria as our pediatric patient, patient 2 was satisfied with the surgical result and concluded that the outcome was good.

## Discussion

Lombok island is part of West Nusa Tenggara Province, Indonesia. Geographically, this province is separated from the mainland of Indonesia, and the distance from Jakarta (the capital of Indonesia) is a 2-h flight. On the other hand, the total population in West Nusa Tenggara is almost 5 million, 65% stay in Lombok island (National Statistics Bureau, 2020). According to the relatively large number of population, many kinds of medical cases are found in this island, including subcutaneous nasal haemangioma. Several patients from this island who have needed advanced or high-technology treatment have refused to be referred to other hospitals outside of Lombok. Repeated consultation is also one of the obstacles on this island. Economic and educational factors influence the decision whether to follow the physician’s recommendation. These factors are reflected by the human development index in West Nusa Tenggara Province, which showed that the island was ranked 29th among 34 provinces in Indonesia [9].

A hemangioma can occur in every part of the body, but the most common area is the head and neck region [3]. Hemangioma around the nasal area occurs in approximately 15.8% of facial hemangioma cases. Subcutaneous hemangioma is the rarest among other types of hemangioma. However, it is common in nasal hemangioma [5, 6]. Nasal hemangioma can result in functional problems, such as nasal obstruction, alteration of nasal valve, ulceration, and destruction of growing nasal cartilage. There is also a psychosocial consequence of nasal hemangioma in growing children [8].

The diagnosis of hemangioma may frequently be based on a patient’s history and clinical examination [10]. However, the imaging workup is necessary to confirm the vascular nature and identify venous, arterial, and lymphatic components as well as the involvement of deeper structures and intracranial connections. A negative result of a CT scan to elucidate the connection between the mass and the central nervous system does not exclude the intracranial connection [10, 11]. Vilanova *et al.* preferred to use magnetic resonance imaging and magnetic resonance angiography to differentiate the type of hemangioma due to its capacity to confirm the diagnosis, determine the extent of the anomaly, classify the lesion appropriately, and document the associated abnormalities [12].

In West Nusa Tenggara Regional Hospital in Lombok, the authors found two cases of nasal dorsum hemangioma in patients of very different ages. The diagnosis of hemangioma was based on physical examination and supported by CT scan to explore the involvement of deeper structures and intracranial connections. The CT scan did not show any connection between the mass and intracranial structures. Biopsy was not performed in both cases, owing to the risk of bleeding; however, postoperative histopathologic examination was done. Histopathologic examination showed a tissue covered by squamous epithelial cells, and there was a proliferation of large arteries inside, which contained erythrocytes (Fig. 3). This histopathologic finding supported the final diagnosis in both cases and was similar to a report in India [13]. Currently, Glut-1 protein (glucose transport protein 1), an immuno-histochemical marker expressed in classic infantile hemangiomas, contributes to diagnosis of hemangioma when the classic histopathologic examination is impossible [14]. Furthermore, Glut-1 can be used to differentiate infantile hemangioma from other vascular anomalies [15].

The treatment options for nasal hemangioma are pharmacological, surgical, or laser interventions [10]. A laser is indicated only for treatment of early lesions to prevent further growth in cases of involuting hemangioma, and it is not helpful for the deeper structures. Corticosteroid is the first-line treatment for nasal hemangioma, and it has few side effects when used as a systemic corticosteroid [8, 16]. The other conservative treatment in the pediatric patient is propranolol. The treatment should be given for at least 5 months; furthermore, the result of this treatment could be partial or good. Nevertheless, there are several side effects of propranolol, especially sleep disturbance [2]. Surgery is necessary for nasal hemangioma to avoid destruction of cartilage. In children, it is recommended to perform surgery between 1 and 2 years old to reduce the likelihood of recurrence and scarring [5]. The present authors did not use lasers, owing to limitations in technology and human resources. Corticosteroid and propranolol were not feasible, owing to long duration of treatment time and side effects; hence, the authors preferred to perform surgery as the choice of treatment.

Several techniques and approaches are used to treat subcutaneous hemangioma. The elliptical or midline elliptical approach is one of the simple methods for managing these cases. There are several advantages and disadvantages of this method. The advantages are a simple and direct approach to the tumor and arteries with a relatively small incision; however, scarring may occur after surgery and could have cosmetic consequences. In a pediatric patient, the authors used an elliptical incision due to the medium size of the tumor. On the other hand, in an adult patient, the authors performed rhinotomy with a lateral incision extended by elliptical incision due to larger mass size and blood vessels. This led to more profuse bleeding in the adult patient. The authors used this approach because of its simplicity and versatility in regard to minimizing tissue removal, skin movement, and incision length. The results of the surgery after 5 months and 3 months in the adult and pediatric patients, respectively, are shown in Fig. 4. Minimal scars were left by the incision; yet, both of the patients said the outcome was good. Furthermore, this result is comparable with the results in other reports [5, 7, 8].

## Conclusion

Because of the limitation of technology and human resources in a remote area such as Lombok, performing surgery for subcutaneous hemangioma in the nasal dorsum is challenging. Nevertheless, in the patients described in this report, the authors provided a good outcome for an adult patient and a pediatric patient through use of an elliptical incision. Furthermore, elliptical incision could be an alternative approach to manage subcutaneous hemangioma in remote areas.

## Supplementary information

**Additional file 1.** Surgical Case Report (SCARE) 2018 checklist.
