# Supplementary material for: Subcutaneous hemangioma on nasal dorsum: a case report
Source: J Med Case Rep. 2020 Aug 13;14:128. doi: 10.1186/s13256-020-02443-4 (PMC7425069; doi:10.1186/s13256-020-02443-4)
Supplement: Supplementary file 1 — Additional file 1. Surgical Case Report (SCARE) 2018 checklist. [file 13256_2020_2443_MOESM1_ESM.docx]

CARE Checklist (2013) of information to include when writing a case report

| Topic | Item | Checklist item description | Reported on Page |
| --- | --- | --- | --- |
| Title | 1 | The words “case report” should be in the title along with the area of focus….. | 1 |
| Key words | 2 | 2 to 5 key words that identify areas covered in this case report | 2 |
| Abstract | 3a | Introduction—what is unique about this case? What does it add to the medical literature? | 2 |
|  | 3b | The main symptoms of the patient and the important clinical findings | 2 |
|  | 3c | The main diagnosis, therapeutics intervention and outcomes | 2 |
|  | 3d | Conclusion—What are the main “take-away” lessons from this case | 2 |
| Introduction | 4 | One or two paragraphs summarizing why this case is unique with reference | 3-4 |
| Patients information | 5a | De-identified demographic information and other patient specific information | 4 |
|  | 5b | Main concerns and symptoms of the patient | 4 |
|  | 5c | Medical, family, and psychosocial history including relevant genetic information (also see timeline) | 4,5 |
|  | 5d | Relevant interventions and their outcomes | 4,5 |
| Clinical findings | 6 | Describe the relevant physical examination (PE) and other significant clinical findings | 4,5 |
| Timeline | 7 | Important information from the patients history organize as timeline | 4,5 |
| Diagnostic | 8a | Diagnostic methods (such as PE, laboratory testing, imaging, surveys) | 4,5,7 |
|  | 8b | Diagnostic challenges (such as access, financial, or cultural) | NA |
|  | 8c | Diagnostic reasoning including other diagnosis considered | 4 |
|  | 8d | Prognostic characteristics (such as staging in oncology)where applicable | NA |
| Therapeutic intervention | 9a | Type of intervention (such as pharmacologic, surgical, preventive, selfcare) | 4,5 |
|  | 9b | Administration of intervention (such as dosage, strength, duration) | 4,5 |
|  | 9c | Change of intervention (with rationale) | NA |
| Follow-up and outcomes | 10a | Clinician and patients-assessed outcomes (when appropriate) | 6,9 |
|  | 10b | Important follow-up diagnostic and other test result | 6,7 |
|  | 10c | Intervention adherence and tolerability (how was this assessed) | NA |
|  | 10d | Adverse and unanticipated events | NA |
| Discussion | 11a | Discussion of the strengths and limitation in your approach to this case | 7-8 |
|  | 11b | Discussion of the relevant medical literature | 7-8 |
|  | 11c | The rationale for conclusion (including assessment of possible causes) | 7,8 |
|  | 11d | The primary “take away” lessons of this case report | 8,9 |
| Patient perspective | 12 | When appropriate the patients should share their perspective on the treatment they received | 8 |
| Informed Consent | 13 | Did the patients give informed consents? Please provide if requested | Yes |
